# Are ambiguity aversion and ambiguity intolerance identical? A neuroeconomics investigation

**DOI:** 10.3389/fpsyg.2014.01550

**Published:** 2015-02-05

**Authors:** Yusuke Tanaka, Junya Fujino, Takashi Ideno, Shigetaka Okubo, Kazuhisa Takemura, Jun Miyata, Ryosaku Kawada, Shinsuke Fujimoto, Manabu Kubota, Akihiko Sasamoto, Kimito Hirose, Hideaki Takeuchi, Hidenao Fukuyama, Toshiya Murai, Hidehiko Takahashi

**Affiliations:** ^1^Department of Psychiatry, Graduate School of Medicine, Kyoto UniversityKyoto, Japan; ^2^Department of Psychology, Waseda UniversityTokyo, Japan; ^3^Research Center for Thinking and Behavioral Judgement, Keio UniversityTokyo, Japan; ^4^Human Brain Research Center, Graduate School of Medicine, Kyoto UniversityKyoto, Japan

**Keywords:** ambiguity aversion, ambiguity intolerance, agreeableness, need for closure, prefrontal cortex, voxel-based morphometry

## Abstract

In recent years, there has been growing interest in understanding a person's reaction to ambiguous situations, and two similar constructs related to ambiguity, “ambiguity aversion” and “ambiguity intolerance,” are defined in different disciplines. In the field of economic decision-making research, “ambiguity aversion” represents a preference for known risks relative to unknown risks. On the other hand, in clinical psychology, “ambiguity intolerance” describes the tendency to perceive ambiguous situations as undesirable. However, it remains unclear whether these two notions derived from different disciplines are identical or not. To clarify this issue, we combined an economic task, psychological questionnaires, and voxel-based morphometry (VBM) of structural brain magnetic resonance imaging (MRI) in a sample of healthy volunteers. The individual ambiguity aversion tendency parameter, as measured by our economic task, was negatively correlated with agreeableness scores on the self-reported version of the Revised NEO Personality Inventory. However, it was not correlated with scores of discomfort with ambiguity, one of the subscales of the Need for Closure Scale. Furthermore, the ambiguity aversion tendency parameter was negatively correlated with gray matter (GM) volume of areas in the lateral prefrontal cortex and parietal cortex, whereas ambiguity intolerance was not correlated with GM volume in any region. Our results suggest that ambiguity aversion, described in decision theory, may not necessarily be identical to ambiguity intolerance, referred to in clinical psychology. Cautious applications of decision theory to clinical neuropsychiatry are recommended.

## Introduction

In recent years, there has been growing interest in understanding a person's reaction to ambiguous situations and two similar concepts related to ambiguity, “ambiguity aversion” and “ambiguity intolerance.” These are described in different disciplines, economics and psychology, respectively. However, it remains unclear whether these two notions derived from different disciplines are identical or not.

In the field of economic decision-making research, ambiguity aversion represents a preference for known risks relative to unknown risks (Ellsberg, 1961; Camerer and Weber, 1992). In economics, “ambiguity” refers to situations in which outcome probabilities are unknown. On the other hand, situations in which people know the precise probabilities of each outcome are referred to as “risk” (Ellsberg, 1961; Camerer and Weber, 1992). To illustrate an example of ambiguity aversion, suppose there are two bowls filled with a mix of 24 blue and red chips each. One bowl has 12 blue and 12 red chips (risky bowl). The composition of the other bowl is unknown to the participants (ambiguous bowl). Participants are asked to select one bowl and told that if a chip with the color blue is drawn, they qualify for a predefined payoff. Most participants would choose the risky bowl, even if its payoff is lower than that of the ambiguous one. Theoretically, the winning probability of both options is the same. For the risky bowl, the probability of drawing the color blue is 0.5. For the ambiguous bowl, the probability of drawing the color blue is unknown, but the winning probability is also 0.5. Nevertheless, previous studies have shown that most individuals shy away from ambiguous options (Ellsberg, 1961; Camerer and Weber, 1992; Levy et al., 2010). Furthermore, previous studies also showed that some individuals have higher ambiguity aversion than others. For example, less optimistic people were reported to have higher ambiguity aversion compared with highly optimistic people (Pulford, 2009).

On the other hand, ambiguity intolerance is a term from the field of psychology. This construct describes the tendency to perceive ambiguous situations as undesirable (Frenkel-Brunswik, 1949), and this is seen in various psychiatric disorders in clinical settings. For example, patients with major depressive disorder often have a “black-or-white thinking,” which is a target of cognitive behavioral therapy (Beck et al., 1979; Andersen and Schwartz, 1992). Furthermore, a previous study reported that the intolerance of (discomfort with) ambiguity subscale of the Need for Closure Scale (NFC) was positively correlated with all aspects of delusion-proneness in non-clinical adults (McKay et al., 2006).

To date, a variety of questionnaires have been developed to measure ambiguity intolerance in psychology. In the psychosocial area of psychology, NFC was developed to estimate individuals' desire for a firm answer to a question and an aversion toward ambiguity (referred to as the need for closure) (Kruglanski et al., 1993). With its relatively high reliability and validity, NFC is widely used in clinical psychology research (e.g., Mancini et al., 2002; McKay et al., 2006). In the context of need for closure, ambiguity intolerance can be assessed by discomfort with ambiguity subscale of NFC. In the subscale, there are items such as “I dislike it when a person's statement could mean many different things” and “I'd rather know bad news than stay in a state of uncertainty.” In the field of psychology, ambiguity often means polysemous or unreliable.

With the advancements of behavioral economics and neuroeconomics, some efforts using these approaches have been made to assess behavioral and psychological problems observed in psychiatric disorders (Sharp et al., 2012). This kind of interdisciplinary research is powerful and useful. However, similar jargon used in the respective fields is not necessarily identical, and thus careful application of the notions between fields is recommended. It seems both promising and natural to apply economic tools to the assessment of ambiguity intolerance in neuropsychiatric disorders. However, studies investigating the relationship between ambiguity aversion and ambiguity intolerance are limited. An earlier study showed that the less tolerant a subject is of ambiguity, the more he prefers to know the odds (Sherman, 1974). Yet, it remains unclear whether these two notions are identical or not.

In recent years, neuroimaging studies have contributed to the development of social neuroscience. While the neural mechanisms that contribute to ambiguous decision-making remain a topic of ongoing investigations, previous functional magnetic resonance imaging (MRI) studies showed the importance of several brain regions such as the dorsolateral prefrontal cortex, anterior cingulate cortex, amygdala, and parietal cortices involved during ambiguous decision-making (Hsu et al., 2005; Huettel et al., 2006; Krain et al., 2006; Bach et al., 2009; Levy et al., 2010). Concerning ambiguity intolerance, a recent functional MRI study reported that intolerance of uncertainty was correlated with insula activation during affective ambiguity (Simmons et al., 2008). However, few studies have endeavored to investigate whether the underlying brain structure is related to ambiguity aversion or ambiguity intolerance in healthy adults. A number of previous studies showed the importance of prefrontal cortices such as the orbitofrontal cortex in decision-making using voxel-based morphometry (VBM) (Ibarretxe-Bilbao et al., 2009; Nakano et al., 2014). However, to the best of our knowledge, no study has directly investigated the relationship between ambiguity aversion and ambiguity intolerance using the VBM technique. Elucidating the neural underpinnings associated with ambiguity aversion and ambiguity intolerance should help us to better understand the relationship of two notions.

In this study, we investigated the relationship between ambiguity aversion and ambiguity intolerance combining an economic task, the NFC questionnaire and VBM of structural MRI. We also administered the self-report version of the Revised NEO Personality Inventory (NEO-PI-R) (Costa and McCrae, 1992), which is one of the most widely-used questionnaires used for assessing broad personality traits. Concerning an economic task, we modified the choice under risk and ambiguity task used in the previous study (Levy et al., 2010). We reasoned that if ambiguity aversion and ambiguity intolerance were identical, these two notions would correlate similarly with fundamental personality traits. A naïve prediction is that ambiguity aversion tendency would be identical to ambiguity intolerance. However, in economics, ambiguity aversion has been estimated by investigating how individuals perceive the odds of the ambiguous options, and thus it could be influenced by the participants' optimism (Pulford, 2009). On the other hand, ambiguity intolerance could be perceived as the extent of discomfort associated with things themselves that are gray instead of black or white. Therefore, we expected that ambiguity aversion would be partially distinct from ambiguity intolerance at behavioral and neural level.

## Materials and methods

### Participants

Thirty-one healthy volunteers were recruited from undergraduate and graduate students. They did not meet the criteria for any psychiatric disorders according to the Structured Clinical Interview for DSM-IV Axis I Disorders (SCID I). They also did not have a history of head trauma, neurological illness, serious medical or surgical illness, or substance abuse. Predicted IQ was estimated with the Japanese Version of the National Adult Reading Test short form (Matsuoka and Kim, 2007). Five participants were excluded from the analyses because one did not choose logical options in a considerable number of trials (i.e., he did not prefer higher amounts relative to lower ones), suggesting that he did not understand the task, and four other participants had behaviors not fitting with our behavioral model. Therefore, data from 26 participants (11 women, 24 right-handed, age: mean = 23.3 [*S.D.* = 6.4] range = 19–40) were analyzed in this study. The estimated mean IQ was 106.3 (*S.D.* = 7.2).

This study was approved by the Committee on Medical Ethics of Kyoto University and carried out in accordance with The Code of Ethics of the World Medical Association. After complete description of the study, written informed consent was obtained from each participant.

### Economic task

**Figure 1:**
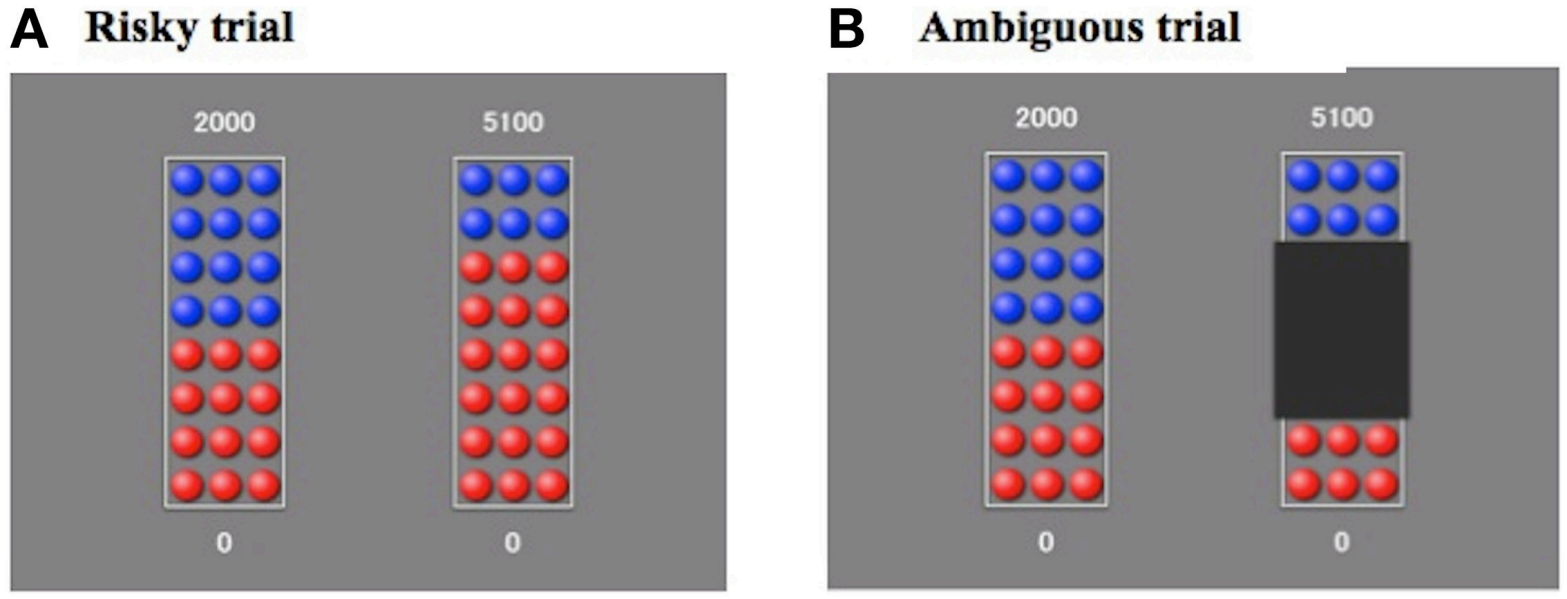
**Experimental design**. Participants were shown two bowls containing 24 blue- and red-colored chips. In each trial, participants were presented with a reference bowl containing 12 blue and 12 red chips on the left side (same throughout the trial). In the risky trials **(A)**, the composition of the variable bowl was visible. In the ambiguous trials **(B)**, part of the variable bowl was hidden by a black occluder. The occluder was always placed over the center of the image. Participants could claim the winning payoff shown beside the color of the drawn chip.

We modified the choice under risk and ambiguity task used in a previous fMRI study (Levy et al., 2010). Participants were told to play a lottery. They were shown two bowls containing 24 blue- and red-colored chips and were told to choose one of the two bowls. From the chosen bowl, one chip was drawn. In each trial, participants were presented with a reference bowl on the left side, which contained 12 blue and 12 red chips (same throughout the trials). As for the other bowl, in half of the trials, the entire bowl was visible. Therefore, participants had information about the ratio of red and blue chips in the bowl (risky trial, Figure 1A) In the other half of the trials, part of the bowl was hidden by a black occluder, which was always placed over the center of the image (ambiguous trial, Figure 1B). Participants had the right to win the payoff next to the color of the drawn chip. As shown in Figure 1, when participants choose the bowl on the left side, if a blue chip is drawn, they will win ￥2000 (about $20), whereas they will win nothing if a red chip is drawn, and when they choose the bowl on the right side, if a blue chip is drawn, they will win ￥5100, whereas they will win nothing if a red chip is drawn. It was explained that their winnings were defined by the sum of the outcomes of three particular trials out of all trials after they had finished (after the task, we debriefed them on the purpose of the experiment and paid the pre-defined participation fee).

There were 60 trials in this task (30 risky trials and 30 ambiguous trials). As for the risky trials, six winning probabilities (1/8, 2/8, 3/8, 5/8, 6/8, 7/8) were used in the variable bowl. There were three ambiguity levels (1/4, 1/2, 3/4) within the ambiguous trials. Five winning amounts (￥1500, ￥2000, ￥3000, ￥5000, ￥10,000) were used at each risk and ambiguity level [5 amounts × (6 risk + 3 ambiguity × 2) conditions = 60 trials]. However, the amounts were varied slightly (±￥100) in each trial in order to prevent participants from developing automatic responses. The winning amount of the reference bowl was always ￥2000. There was no time limit for choosing options in each trial. No feedback of the outcome after each choice was provided. The interval between successive trials was 2.5 s. The experiment was presented using E-Prime software (Psychology Software Tools, Inc., Pittsburgh, PA, USA). The order of the trials was randomized across participants. In addition, the winning colors were mixed between participants.

### Measures

#### Need for closure scale

The Need for Closure Scale (NFC) measures an individual's tendency to obtain firm answers and to avoid ambiguity (Kruglanski et al., 1993). NFC is composed of 42 items and contains five subscales (preference for order, preference for predictability, decisiveness, discomfort with ambiguity, and closed-mindedness). Participants are asked to indicate how much they agree with a statement by responding using a 6-point scale ranging from 1 (strongly disagree) to 6 (strongly agree). Higher scores indicate greater need for closure. To estimate ambiguity intolerance in the context of need for closure, we used the discomfort with ambiguity subscale. This subscale consists of nine items, such as “I dislike it when a person's statement could mean many different things” and “I'd rather know bad news than stay in a state of uncertainty.” Other subscales are described in Appendix 1 in Supplementary Material.

#### Revised NEO personality inventory

To assess the Big Five personality factors, we administered the Japanese version of the Revised NEO Personality Inventory (NEO-PI-R) (Costa and McCrae, 1992; Shimonaka et al., 1997). This self-report questionnaire is composed of 240 items and contains five dimensional scales (Neuroticism, Extraversion, Openness, Agreeableness, and Conscientiousness) corresponding to a five-factor model of personality trait. In each item, participants are asked to mark from 0 (strongly disagree) to 4 (strongly agree). NEO-PI-R results are presented as T scores with a mean of 50 and a *S.D.* of 10.

### MRI acquisition and pre-processing

All participants underwent MRI scans by 3-T whole-body scanner equipped with an 8-channel phased-array head coil (Trio, Siemens, Erlangen, Germany). The scanning parameters of the T1-weighted three-dimensional magnetization-prepared rapid gradient-echo (3D-MPRAGE) sequence were as follows: TR = 2000 ms, TE = 4.38 ms, TI = 990 ms, FOV = 225 × 240 mm, matrix = 240 × 256, resolution = 0.9375 × 0.9375 × 1.0 mm^3^, and 208 total axial sections without intersection gaps.

MRI data were processed using SPM8 (Wellcome Trust Center for Neuroimaging, London, UK), and the VBM8 toolbox (http://dbm.neuro.uni-jena.de/vbm/) running on Matlab (MathWorks, Natick, MA, USA). In brief, all images were tissue classified and spatially normalized to the same stereotaxic space by using the diffeomorphic anatomical registration through exponentiated Lie algebra (DARTEL) algorithm (Ashburner, 2007). The voxel values of segmented and normalized gray matter (GM) images were modulated by the Jacobian determinants obtained from non-linear normalization steps. We used the default parameters of the VBM8 toolbox, but, as an exception, the ICBM space template for East Asian brains was applied for affine regularization. Finally, the resultant GM images were smoothed with Gaussian kernels of 8 mm full width at half maximum, on which all analysis were performed.

### Data analyses

#### Behavioral analysis

Individual ambiguity aversion tendencies were estimated according to a previous study (Levy et al., 2010). First, the subjective value (SV) of each option was modeled using a power function (Kahneman and Tversky, 1979):

$$SV=\left[p-\beta \left(\frac{A}{2}\right)\right]\times V^{\alpha }$$

where *A* is the ambiguity level, *p* is the objective probability, *V* is the winning amount, α is the individual risk aversion parameter, and β is the ambiguity aversion parameter. Note that *A* is 0 for the risky bowls (including the reference bowl), and *p* is 1/2 for the ambiguous bowls.

Then, using maximum likelihood, we fitted the choice results of each participant with the following formula:

$$P_{v}=\frac{1}{1+e^{\gamma (SV_{F}\;-\;SV_{v})}}$$

where *P*_*v*_ is the probability that the subject selected the variable bowl, *SV*_*F*_ and *SV*_*v*_ are the *SV* of the fixed and variable options, respectively, and γ is the slope of the logistic function, a subject-specific parameter. For non-linear optimization, we used the sequential quadratic programming method implemented in SPSS 21 (IBM, Armonk, NY, USA) with 27 starting positions. The initial values (α: 0.3, 0.75, 1.2; β: 0, 0.5, 1; γ: 0.08, 1, 12) were selected based on the results of a previous study (Levy et al., 2010). Because the data of four participants failed to converge, they were excluded.

An ambiguity-averse participant would show β > 0, and an ambiguity-seeking participant would show β < 0. As for risk, a risk-averse participant would show α < 1, and a risk-seeking participant would show α > 1.

To investigate the relationship between individual ambiguity aversion tendency and individual ambiguity intolerance tendency, we performed a correlational analysis between individual ambiguity aversion parameter and the scores of discomfort with ambiguity. Correlation analyses were also performed between individual ambiguity aversion parameter and each T score of five domains of NEO-PI-R. Similarly, we also performed correlational analyses between the scores of discomfort with ambiguity and each T score of five domains of NEO-PI-R. Results were considered statistically significant at *p* < 0.05. Because of the exploratory nature of the analysis, multiple comparison correction was not performed on these correlational analyses.

#### Image analysis

To explore the brain region that was correlated with ambiguity aversion parameter and ambiguity intolerance tendency throughout the whole brain, we performed multiple regression analysis separately using the general linear model framework in SPM8. Age, gender and predicted IQ were entered into the model as covariates of no interest. Because the images were modulated for non-linear warping only, intracranial volume was not included as nuisance covariate (http://dbm.neuro.uni-jena.de/vbm/segmentation/modulation/). A statistical threshold of *p* < 0.001 (uncorrected) with an extent threshold of 100 voxels was applied. The choice of these thresholds was based on exploratory data analyses and on effect size considerations derived from previous VBM studies (Kubota et al., 2011; Sasamoto et al., 2011).

We interpreted the anatomical location of the clusters by consulting the Anatomic Automatic Labeling toolbox (Tzourio-Mazoyer et al., 2002), the xjView toolbox (http://www.alivelearn.net/xjview8/), and neuroanatomy atlas books (Talairach and Tournoux, 1988; Duvernoy, 1991).

## Results

Behavioral data from the 26 analyzed participants fitted well with our behavioral model (*R*^2^: mean = 0.71 [*S.D.* = 0.12], range = 0.38–0.89). Participants varied widely in the degree of risk and ambiguity aversions (α: mean = 0.45 [*S.D.* = 0.33], range = 0.02–1.27; β: mean = 0.61 [*S.D.* = 0.25], range = 0.22–1.10). However, most subjects exhibited both risk and ambiguity aversion. In our study, the reference bowl was always presented on the left side. Nevertheless, participants did not follow a strategy of choosing only one side. Mean response time in ambiguous trials was slower than that in risky trials (risk: mean = 2.05 s [*S.D.* = 0.82]; ambiguity: mean = 2.57 s [*S.D.* = 1.07], *p* < 0.01).

The mean scores of discomfort with ambiguity were 34.6 [*S.D.* = 6.0], range = 21–44 (results of other subscales of NFC are shown in Supplementary Table S1). The mean ± *S.D.* [range] of the T score of five domains of NEO-PI-R were as follows: Neuroticism: 49.5 ± 11.1 [29–72]; Extraversion: 49.4 ± 11.5 [19–79]; Openness: 50.0 ± 9.2 [33–67]; Agreeableness: 52.5 ± 10.5 [31–71]; Conscientiousness: 53.1 ± 11.1 [34–72].

In the five domains of NEO-PI-R, a negative correlation was found between the Agreeableness domain and ambiguity aversion parameter (*r* = −0.56, *p* < 0.01) [the other four domains were not significantly correlated with ambiguity aversion parameter (all, *p* > 0.07)]. As for the scores of discomfort with ambiguity, they were positively correlated with the Neuroticism domain (*r* = 0.44, *p* = 0.03) and negatively correlated with the Extraversion domain (*r* = −0.48, *p* = 0.01) [the other three domains were not significantly correlated with the scores of discomfort with ambiguity (all, *p* > 0.33)]. However, the scores of discomfort with ambiguity were not correlated with individual ambiguity aversion parameter (*r* = −0.13, *p* = 0.53) [there was also no significant correlation between individual ambiguity aversion parameter and any other subscales of NFC (all, *p* > 0.20)].

**Figure 2:**
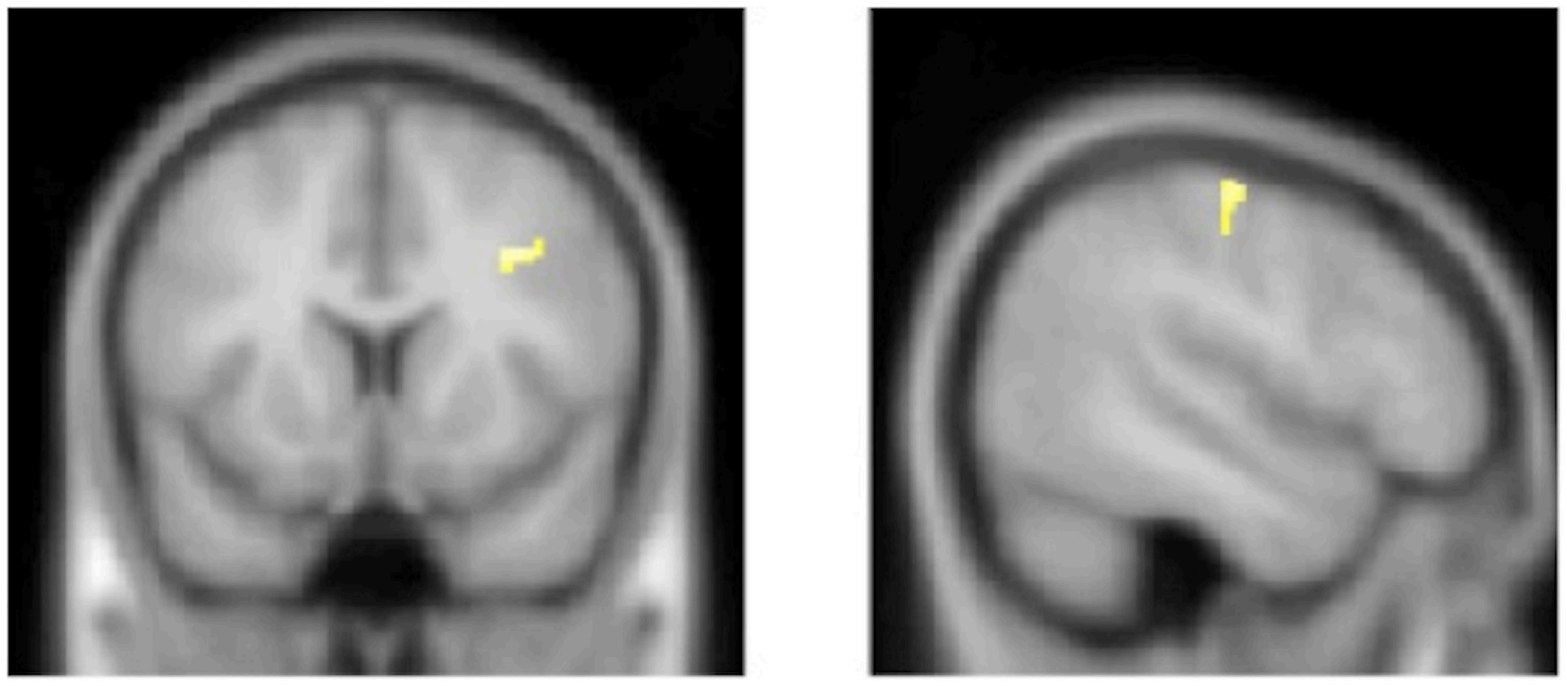
**Significant clusters negatively correlated with individual ambiguity aversion parameter**. Clusters negatively correlated with individual ambiguity aversion parameter are shown. Height and extent thresholds were set at *p* < 0.001, uncorrected and *k* = 100 voxels. The clusters including the right middle frontal gyrus extending into right posterior inferior gyrus, and the right postcentral gyrus were negatively correlated with individual ambiguity aversion parameter.

Next, we conducted a separate correlation analysis between GM volume and individual ambiguity aversion parameter and between GM volume and the scores of discomfort with ambiguity. The individual ambiguity aversion parameter was negatively correlated with GM volume in the right middle frontal gyrus (MFG) extending into the right posterior inferior frontal gyrus (pIFG), and the right postcentral gyrus (PCG) [No significant clusters emerged with positive correlation with individual ambiguity aversion parameters.] (Table 1, Figure 2.). On the other hand, scores of discomfort with ambiguity were not correlated with the GM volume in any region.

**Table 1:** **Brain regions negatively correlated with individual ambiguity aversion parameters**.

**Brain region**	**H**	**Coordinates (mm)**			***t***	**Cluster (voxel)**
		***x***	***y***	***z***		
Middle frontal gyrus and posterior inferior frontal gyrus	R	36	11	36	4.68	100
Postcentral gyrus	R	54	−12	54	4.86	179

P < 0.001, uncorrected and k = 100.

MNI coordinates and t-values are provided for the local voxel maximum of the respective cluster.

Abbreviations: Lt, left; Rt, right; H, hemisphere.

## Discussion

In this study, we investigated the relationship between ambiguity aversion and ambiguity intolerance at both the behavioral and neural level. The individual ambiguity aversion tendency parameter was negatively correlated with agreeableness scores on NEO-PI-R, but it was not correlated with scores of discomfort with ambiguity. Furthermore, ambiguity aversion tendency was negatively correlated with GM volume in the right MFG extending into the right pIFG, and the right PCG, whereas scores of discomfort with ambiguity were not correlated with GM volume in any region. These results suggest that ambiguity aversion and ambiguity intolerance are not necessarily identical.

In our economic task, participants varied widely in their degrees of ambiguity aversion. However, most participants showed ambiguity aversion, which is consistent with previous studies (Ellsberg, 1961; Camerer and Weber, 1992; Hsu et al., 2005; Levy et al., 2010). Concerning the response time for each probability condition, the response time in ambiguous trials was slower than that in risky trials. This result is also in line with a previous study (Bach et al., 2011).

In the correlation analysis, the ambiguity aversion parameter did not correlate with scores of discomfort with ambiguity. In the field of psychology, “ambiguity” means not only imprecision or uncertainty but also the presence of multiple meanings (Werman, 1979). Indeed, the items of discomfort with ambiguity subscale imply a distaste for polysemous situations, such as “I dislike it when a person's statement could mean many different things.” On the other hand, ambiguity aversion is constrained to situations in which the probabilities of future outcomes are unknown. Therefore, ambiguity intolerance might be much broader than ambiguity aversion. Accordingly, people who are ambiguity intolerant may not necessarily be ambiguity averse if their intolerance is not focused on an economically ambiguous situation. We did not find significant associations between ambiguity aversion and ambiguity intolerance, while a previous study reported that the less ambiguity tolerant a subject is, the more he prefers to know the odds (Sherman, 1974). This discrepancy might be due to characteristics of the participants or the applied economic task design. The previous study was limited to male undergraduate students, but we recruited participants from both genders. As for the task design, the earlier study performed a single trial for measuring individual ambiguity aversion tendency, while our study performed 60 trials for a more accurate evaluation.

In the five domains of NEO-PI-R, there was a negative correlation between the Agreeableness domain and ambiguity aversion parameter. A possible interpretation is as follows. Individuals with high agreeableness scores are generally optimistic and believe that most people are honest and trustworthy (Costa and McCrae, 1992). Like their optimistic view of human nature, they might have an optimistic prediction of the probabilities of the ambiguous bowl. This speculation is in line with the previous study that showed highly optimistic people less frequently shy away from ambiguous options (Pulford, 2009). On the other hand, the scores of discomfort with ambiguity were positively correlated with the Neuroticism domain and negatively correlated with the Extraversion domain. The present result, that ambiguity aversion was associated with a different personality domain from those correlated with ambiguity intolerance, is in line with the assumption that these two notions are not necessarily identical. Ambiguity intolerance describes the tendency to perceive ambiguous situations as undesirable (Frenkel-Brunswik, 1949), which is seen in various psychiatric disorders such as major depressive disorder. Therefore, it is reasonable that the scores of discomfort with ambiguity were positively correlated with the Neuroticism domain that includes anxiety, depression, and vulnerability to stress. The negative correlation between the discomfort with ambiguity and the Extraversion domain was also consistent with the previous study, which showed that individuals who were sociable, talkative, and sought social activities were more ambiguity tolerant (Caligiuri and Tarique, 2012).

Interestingly, the individual ambiguity aversion parameter was negatively correlated with GM volume in the right MFG/pIFG and right PCG. As for the right MFG/pIFG, these areas in the prefrontal cortex play roles in higher cognition and behavioral planning (Lee et al., 2007; Sakagami and Watanabe, 2007; Barbey and Patterson, 2011), which are important for ambiguous decision-making. Indeed, the lateral prefrontal cortex, including the MFG and IFG, was frequently reported as being activated in functional neuroimaging studies related to ambiguous decision-making (Krain et al., 2006; Bach et al., 2009; Lopez-Paniagua and Seger, 2013). Furthermore, a previous study reported that the brain activation of areas (MNI coordinates; 39, 16, 33) near the above-mentioned cluster in the right MFG/pIFG during a task related to ambiguous decision-making was correlated with an individual's ambiguity preference parameter, and the authors proposed that this region implements contextual analysis and inhibits impulsive responses (Huettel et al., 2006). In line with this notion, previous VBM studies showed that GM volume reduction in the lateral prefrontal cortex was associated with impulsivity behavior (Cho et al., 2013; Qiu et al., 2013). Taken together, our results are in line with the previous neuroimaging studies, suggesting that volumetric differences in the right lateral prefrontal cortex can also account for the inter-individual variability in ambiguity aversion tendency.

We also found a negative correlation between ambiguity aversion parameter and the cluster including the right PCG that plays a key role in sensory processing (Keysers et al., 2010). Multiple areas in the parietal cortex including the PCG were also reported as being activated in the functional neuroimaging studies related to ambiguous decision-making (Krain et al., 2006; Bach et al., 2011), suggesting that the parietal cortex has roles in the process of resolving ambiguity. Ambiguous decision-making is quite complex because of a lack of information about outcome probabilities, and sensory integration is considered to be involved in ambiguous decision-making (Bach et al., 2009). Our results are in line with this notion, supporting the theory that the parietal cortex is crucial in ambiguous decision-making. Recently, the PCG has been considered to not only encode bodily sensations but also play a key role in social perception (Hooker et al., 2008; Keysers et al., 2010). This area should be further explored in the future in terms of its role in decision-making processes.

On the other hand, the neural basis of ambiguity intolerance remains obscure, as we did not find any significant brain regions that were correlated with the scores of discomfort with ambiguity. This may be because the neural basis for ambiguity intolerance depends on more general mechanisms underlying a broad range of cognitive and social abilities, or because the relationship between ambiguity intolerance and morphological difference is too subtle to find in a sample of this study. However, our null findings should be interpreted with extreme caution, as the sample size of our study is small. In addition, the variation of the scores of discomfort with ambiguity was small compared to that of the ambiguity aversion parameter. This might cause the difference in the VBM results of these two notions. Taken together, it is clear that we need to be careful when interpreting the relationship between two notions based on the present VBM results.

There are several limitations to this study. First, the scale of our study was modest for understanding individual differences. Second, we did not perform the economic task using real money. Third, we treated ambiguity intolerance in the context of need for closure using the discomfort with ambiguity subscale of NFC, although there are a number of questionnaires that measure ambiguity intolerance (e.g., Eysenck, 1954; Budner, 1962). Although discomfort with ambiguity subscale of NFC was reported to correlate with the Intolerance of Ambiguity Scale by Eysenck (1954; Webster and Kruglanski, 1994), caution should be exercised in generalizing the present results. Further research will be needed to replicate the present results utilizing multiple instruments addressing ambiguity intolerance in a larger number of subjects. Fourth, a correction for multiple comparisons was not applied during the correlation analyses with NEO-PI-R. Fifth, we measured ambiguity attitudes with our economic task that included only the domain of gains. Previous studies showed that individuals reacted to a particular choice in a different manner whether it was presented as a gain or as a loss (referred to as “framing effect”) (Tversky and Kahneman, 1981; De Martino et al., 2006). In the future, we should examine a person's decisions under conditions of ambiguity both in the domain of gains and in the domain of losses. Finally, in the brain structures, the focus of this study was restricted to the GM volumes. Of course, white matter (WM) connectivity also has key roles in the neural basis of decision-making. Future research utilizing multimodal MRI will need to investigate the interplay between GM and WM on ambiguous decision-making for better understanding of the neural underpinnings associated with ambiguity aversion and ambiguity intolerance.

In conclusion, we compared ambiguity aversion described in decision theory with ambiguity intolerance referred to in clinical psychology at both a behavioral and a neural level. Our findings suggest that ambiguity aversion and ambiguity intolerance are not necessarily identical. Although an interdisciplinary approach to applying decision theory to clinical neuropsychiatry should be expected to be both promising and ultimately useful, careful application is recommended.

### Conflict of interest statement

The Guest Associate Editor Mitsuhiro Okada declares that, despite having collaborated with author Shigetaka Okubo, the review process was handled objectively and no conflict of interest exists. The authors declare that the research was conducted in the absence of any commercial or financial relationships that could be construed as a potential conflict of interest.
