# Supplementary material for: Are ambiguity aversion and ambiguity intolerance identical? A neuroeconomics investigation
Source: Front Psychol. 2015 Feb 5;5:1550. doi: 10.3389/fpsyg.2014.01550 (PMC4318272; doi:10.3389/fpsyg.2014.01550)
Supplement: Supplementary file 1 [file Table1.DOCX]

***Supplementary Material***

**Are ambiguity aversion and ambiguity intolerance identical? A neuroeconomics investigation**

**Yusuke Tanaka^1†^, Junya Fujino^1†^, Takashi Ideno^2^, Shigetaka Okubo^2,3^, Kazuhisa Takemura^2^, Jun Miyata^1^, Ryosaku Kawada^1^, Shinsuke Fujimoto^1^, Manabu Kubota^1^, Akihiko Sasamoto^1^, Kimito Hirose^1^, Hideaki Takeuchi^1^, Hidenao Fukuyama^4^, Toshiya Murai^1^, Hidehiko Takahashi^1^***

^1^Department of Psychiatry, Graduate School of Medicine, Kyoto University, Kyoto, Japan

^2^Department of Psychology, Waseda University, Tokyo, Japan

^3^Research Center for Thinking and Behavioral Judgement, Keio University, Tokyo, Japan

^4^Human Brain Research Center, Graduate School of Medicine, Kyoto University, Kyoto, Japan

^†^ These authors have contributed equally to this work.

*** Correspondence:** Hidehiko Takahashi, Department of Psychiatry, Graduate School of Medicine, Kyoto University, Shogoin-Kawaharacho 54, Kyoto 606-8507, Japan.

[hidehiko@kuhp.kyoto-u.ac.jp](mailto:hidehiko@kuhp.kyoto-u.ac.jp)

**Appendix 1**

**Additional information on the subscales of the Need for Closure Scale (NFC)**

NFC (Kruglanski et al., 1993) is composed of 42 items and contains five facets: preference for order, preference for predictability, decisiveness, discomfort with ambiguity, and closed-mindedness. Preference for order consists of 10 items (e.g. “I think that having clear rules and order at work is essential for success”), preference for predictability consists of 8 items (e.g. “I don’t like to go into a situation without knowing what can I expect from it''), decisiveness consists of 7 items (e.g. “I usually make important decisions quickly and confidently”), discomfort with ambiguity consists of 9 items (e.g. “I'd rather know bad news than stay in a state of uncertainty”), and closed-mindedness consists of 8 items (e.g. “I do not usually consult many different opinions before forming my own view”).

**Supplementary Table**

**Suplementary Table S1. Scores of each NFCS subscale**

| NFCS | mean ± S.D. [range] |
| --- | --- |
| preference for order | 37.5 ± 5.4 [26-47] |
| preference for predictability | 28.8 ± 5.1 [18-41] |
| decisiveness | 19.8 ± 5.4 [10-31] |
| discomfort with ambiguity | 34.6 ± 6.0 [21-44] |
| closed-mindedness | 25.2 ± 4.1 [18-35] |

**References**

Kruglanski, A.W., Webster, D.M., and Klem, A. (1993). Motivated resistance and openness to persuasion in the presence or absence of prior information. *J Pers Soc Psychol* 65**,** 861-876.
